# Factors affecting R01 grant funding among academic neurosurgeons over the last decade

**DOI:** 10.1016/j.amsu.2020.06.002

**Published:** 2020-06-04

**Authors:** Joshua A. Cuoco, Brendan J. Klein, Ayesha Kar, Hailey L. Gosnell, Evin L. Guilliams, Michael J. Benko, Lisa S. Apfel, John J. Entwistle, Eric A. Marvin, Mark R. Witcher

**Affiliations:** a Carilion Clinic, Section of Neurosurgery, 1906 Belleview Ave, Roanoke, VA, 24014, USA; b Virginia Tech Carilion School of Medicine, 2 Riverside Circle, Roanoke, VA, 24016, USA; c Virginia Tech School of Neuroscience, Blacksburg, VA, 24061, USA

**Keywords:** Academic medicine, Medical education, Osteopathic physician, Allopathic physician, Neurosurgery, R01, Research Project Grant, NIH, National Institutes of Health, MD, Doctor of Allopathic medicine, DO, Doctor of Osteopathic medicine, PhD, Doctor of Philosophy, MPH, Master of Public Health, MBA, Master of Business Administration, MHS, Master of Health Science, MS, Master of Science, MA, Master of Arts

## Abstract

**Background:**

Recent studies have reported a gender and medical degree disparity for those receiving Research Project Grants in surgical specialties. The aim of the present study is to analyze factors among academics neurosurgeons that correlate to higher amounts of R01 grant monies awarded.

**Materials and methods:**

The National Institutes of Health Research Portfolio Online Reporting Tools Expenditures and Results database was queried for neurosurgery funding between 2008 and 2018. Grant recipients were categorized among type of degree, secondary degree(s), professorship, gender, and h - index. Statistical analysis was performed.

**Results:**

The National Institutes of Health awarded 480 R01 grants totaling $182,482,644 to 81 allopathic neurosurgeons between 2008 and 2018. No osteopathic neurosurgeons were awarded an R01 grant during this timeframe. There was a significant difference for type of professorship on the total awarded amount at the p < 0.05 level for the three types of professorship [F (2,78) = 4.85, p < 0.01)]. There was a significant difference for magnitude of h – index on total R01 monies (p < 0.00001). Males accounted for the majority of R01 monies (93.99%); however, no significant difference between average amount awarded and gender was identified (p = 0.86). A secondary degree was without significant difference for R01 amount awarded (p = 0.75).

**Conclusions:**

The present study establishes a medical degree disparity for academic neurosurgeons who receive an R01 grant. Statistically significant factors found to affect amount of R01 grant monies awarded were limited to type of professorship and magnitude of h – index.

## Highlights

• $182,482,644 in R01 monies was awarded to allopathic neurosurgeons between 2008 and 2018.
• No osteopathic neurosurgeons were awarded an R01 grant between 2008 and 2018.
• There was a significant difference for type of professorship on total R01 monies.
• There was a significant difference for magnitude of h – index on total R01 monies.

## 1 Introduction

A primary objective of career development for the physician-scientist is acquiring adequate funding to sustain their research endeavors. In expectation of this career milestone, junior investigators aim to develop an ample body of research experience, national presentations, and publication content for federal research monies and, ultimately, contend for a Research Project Grant (R01) award by the National Institutes of Health (NIH). The R01 is the original and longest-standing grant provided by the NIH. As such, acquiring an R01 grant is considered a prestigious achievement for the physician-scientist and a milestone for progression in the world of academia. However, receiving an R01 award is not a precondition for a prosperous research career nor is the NIH the only body of extramural funding that supplies such awards. Nevertheless, the R01 is still a well sought after research grant due to its worldwide recognition, prestige and known standardized rigorous process by which evaluation and decisions are made.

Recent studies have reported a gender and degree disparity for those receiving R01 grants in surgical specialties [1,2]. Berg et al. reported a gender and medical degree disparity for those receiving an NIH R01 grant in the specialty of general surgery [1]. Moreover, Eloy et al. found that male faculty members in otolaryngology departments have higher NIH grant monies compared to their female counterparts [2]. However, to our knowledge, no study to date has investigated if similar disparities exist in the subspecialty of neurosurgery. The aim of the present study is to analyze factors among academics neurosurgeons that correlate to higher amount of NIH R01 grant monies awarded. Moreover, we investigate for potential disparities between those receiving an NIH R01 grant in the subspecialty of neurosurgery.

## 2 Materials and Methods

We queried the NIH Research Portfolio Online Reporting Tools Expenditures and Results database (www.report.nih.gov) search engine for “neurosurgery” as a key word to examine R01 grants issued between 2008 and 2018. An Internet search was used to identify the credentials of each grant recipient. Primary investigators who completed a neurosurgical residency were included in our cohort. All other specialties and sub-specialties were excluded from the analysis. Grant recipients were then categorized among type of primary medical degree (allopathic (MD) or osteopathic (DO)), additional secondary degree(s) (Doctor of Philosophy (PhD), Master of Public Health (MPH), Master of Business Administration (MBA), Master of Health Science (MHS), Master of Science (MS), or Master of Arts (MA)), professorship (full professor, associate professor, or assistant professor), gender and h -index. These details were gathered via an Internet search for the grant recipient. The Scopus database (www.scopus.com) was searched to determine each grant recipient's h – index. Additionally, the total number of grants funded and total awarded amounts were recorded based on our query. Data was analyzed with statistical tests including Mann-Whitney U-Tests, analysis of variance, and post-hoc Tukey's honestly significant difference.

## 3 Results

Our query identified 276 R01 grant recipients between 2008 and 2018. Non-medical doctors (PhD only) accounted for 56.16% (155 of 276) of all grant recipients. Allopathic physicians accounted for the remaining 43.84% (121 of 276) of grant recipients. No osteopathic physicians were awarded an R01 grant between 2008 through 2018 based on our query. An Internet search of the biography of the 121 allopathic physician grant recipients revealed only 81 of the 121 recipients to be neurosurgeons. As such, allopathic neurosurgeons represented 29.35% (81 of 276) of all grant recipients. Within this cohort, 38.27% (31 of 81) of neurosurgeons held a dual-degree. Specifically, 29.63% (24 of 81) held a PhD, 6.17% (5 of 81) held a MS, 4.94% (4 of 81) held a MBA, 1.23% (1 of 81) held a MPH, 1.23% (1 of 81) held a MA, and 1.23% (1 of 81) held a MHS. Furthermore, 77.78% (63 of 81) held full professorship, 9.88% (8 of 81) held associate professorship, and 12.35% (10 of 81) held assistant professorship. Males accounted for 91.36% (74 of 81) of neurosurgeon grant recipients whereas females accounted for the remaining 8.64% (7 of 81).

**Fig. 1:**
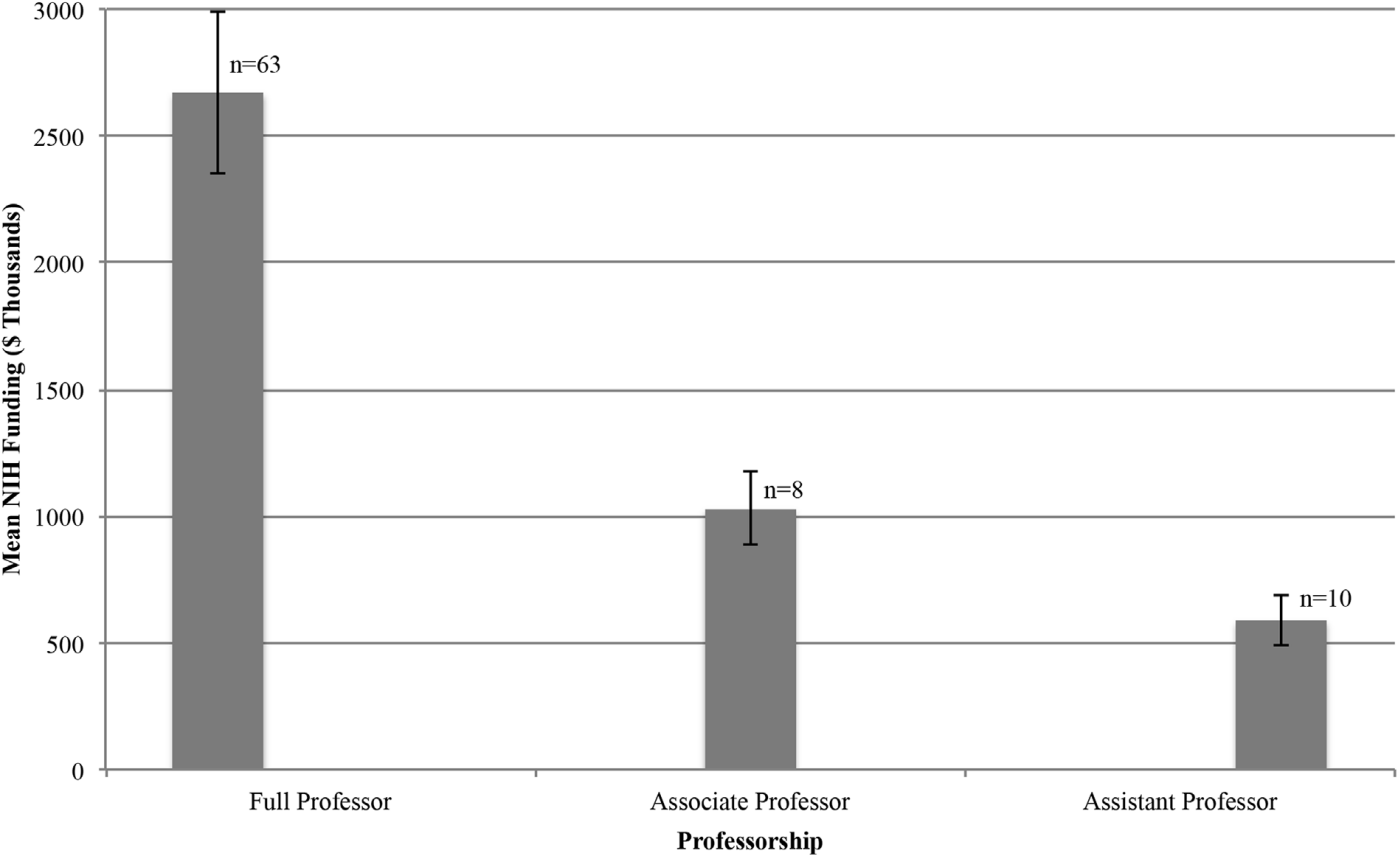
Mean NIH funding among academic neurosurgeons stratified by professorship. Error bars represent standard error of the mean.

**Fig. 2:**
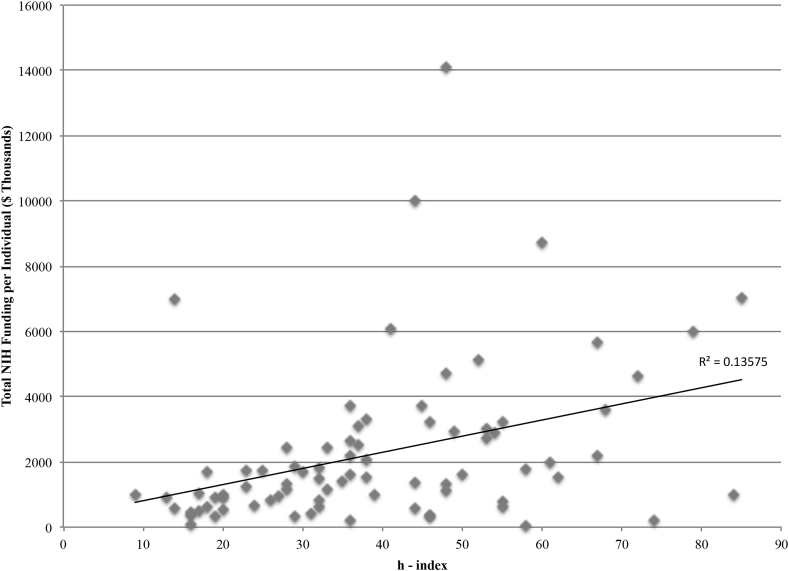
Total NIH funding among academic neurosurgeons stratified by h-index.

**Fig. 3:**
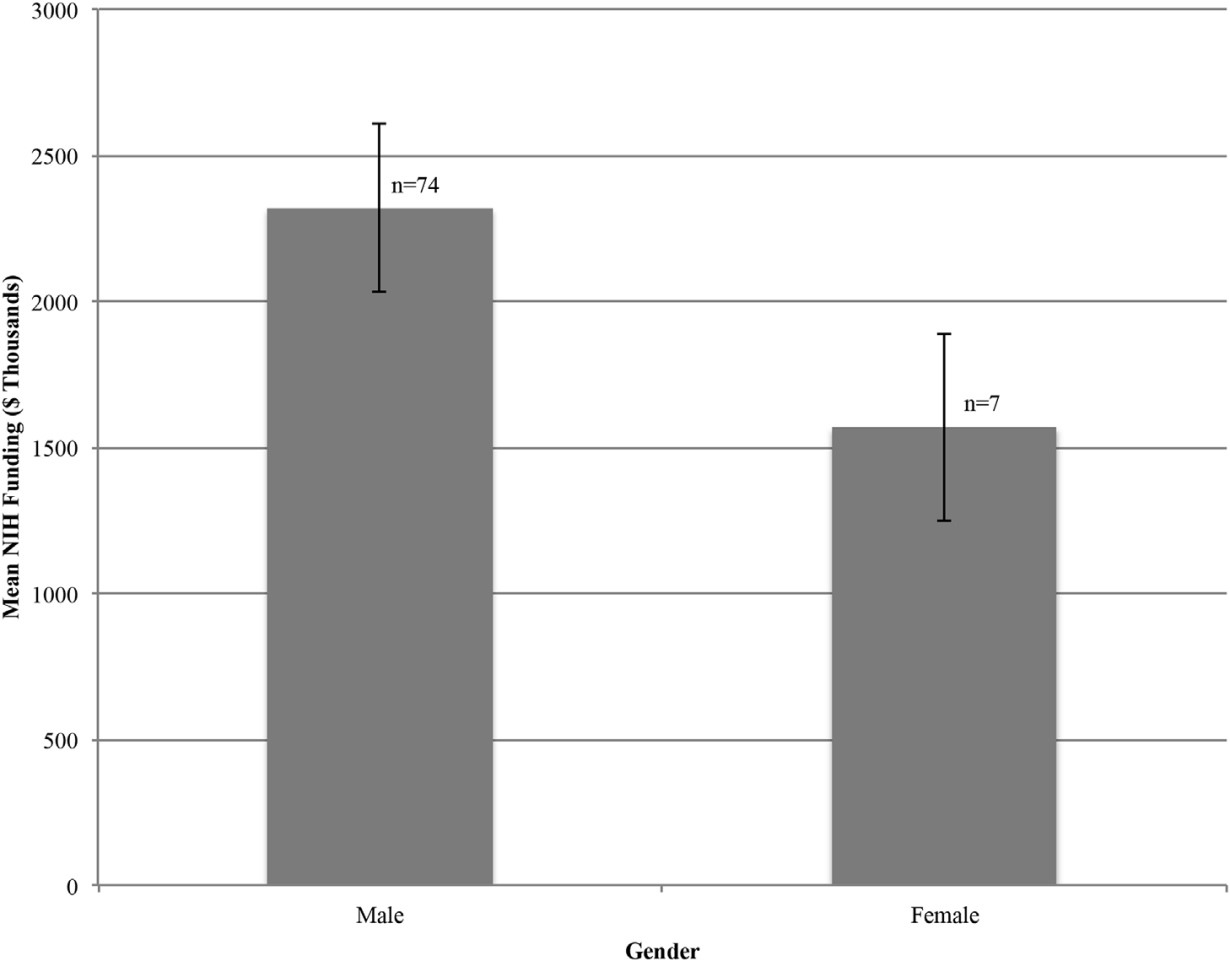
Mean NIH funding among academic neurosurgeons stratified by gender. Error bars represent standard error of the mean.

A total of 480 R01 grants were identified. Total R01 grant award monies in the present cohort were calculated to be $182,483,644 (mean $2,252,884; median $1,512,213; range $50,000 - $14,115,363). Advanced statistics were utilized to examine factors that may impact R01 monies awarded. Full professors, associate professors, and assistant professors received an average of $2,672,386, $1,027,613, and $590,245 R01 grant monies, respectively (Fig. 1). There was a significant difference for type of professorship on the total awarded amount at the p < 0.05 level for the three types of professorship [F (2,78) = 4.85, p < 0.01)]. Post-hoc comparisons using the Tukey's honestly significant difference test indicated statistical significance between total awarded monies between the full professorship and assistant professorship group (p < 0.02) but did not demonstrate statistical significance between the full and associate professorship groups (p = 0.14) or associate and assistant professorship groups (p = 0.90). There was a significant difference for magnitude of h – index on total R01 award monies (Mann-Whitney U-Tests, p < 0.00001) (Fig. 2). Individuals with a secondary degree received an average of $2,445,502 compared to an average of $2,133,461 for those without a secondary degree. However, a secondary degree was without statistical significant difference for R01 amount awarded (Mann-Whitney U-Tests, p = 0.75). Moreover, an additional doctoral degree (PhD) was without statistical significant difference for R01 amount awarded (Mann-Whitney U-Tests, p = 0.74). Although male neurosurgeons accounted for 93.99% of all 10.13039/100000002NIH R01 award monies there was no statistically significant difference between the average amount awarded and gender (Mann-Whitney U-Tests, p = 0.86) (Fig. 3).

## 4 Discussion

The present study, to our knowledge, is the first to investigate if certain innate and acquired factors of the academic neurosurgeon correlate to successful R01 awards and R01 monies. Our analyses demonstrated a medical degree disparity for academic neurosurgeons who receive an R01 grant. Indeed, no osteopathic neurosurgeons received an NIH R01 grant over the past decade despite the fact that osteopathic neurosurgeons represented 2.0% of the 5530 neurosurgeons in the United States of America in 2017 [3]. While it remains unclear why such a disparity exists in the context of NIH R01 grants, prior reports have begun to unveil similar incongruences [[4], [5], [6], [7], [8], [9]]. Hoehmann et al. investigated osteopathic representation on the editorial boards of 50 continuing neurosurgical journals [8]. The authors found osteopathic neurosurgeons represented 1 (0.04%) of the 2286 editorial positions [8]. These data established that the percentage of United States osteopathic neurosurgeons (2.0%) fulfilling editorial positions of neurosurgical journals was not proportional to the percentage of editorial positions they hold on neurosurgical journals (0.04%). Moreover, Cuoco et al. examined the number of osteopathic neurosurgeons fulfilling authorship positions in Journal of Neurosurgery Publishing Group journals since 1944 [9]. The authors found that osteopathic neurosurgeons represented 153 (0.15%) of the 105,492 authorship positions available [9]. These data established that the percentage of United States osteopathic neurosurgeons (2.0%) was not proportional to the percentage of authorship positions they hold in Journal of Neurosurgery Publishing Group journals (0.15%). The absence of osteopathic neurosurgeon representation on NIH R01 awards over the past decade may be attributable to numerous factors that begin with significant differences between allopathic and osteopathic medical schools. Compared to their allopathic counterparts, osteopathic medical schools received 800 times less funding from the NIH in 2011, exhibit a lack of focus on research endeavors reported by their student body, and lack available dual-degree programs to spark a career as a physician-scientist [9]. Indeed, these data provide a foundational basis for the lack of proportionality between the percentages of practicing osteopathic neurosurgeons and R01 awards. Furthermore, all Accreditation Council for Graduate Medical Education neurosurgery residency programs in the United States of America (traditionally training allopathic residents) focus on preparing residents for careers in academic neurosurgery with 1–2 years of dedicated research within a 7-year program. This is in contrast to now extinct American Osteopathic Association neurosurgery residency programs (training osteopathic residents), which lacked a focus on research. With the recent merging of Accreditation Council for Graduate Medical Education and American Osteopathic Association residency programs, all neurosurgical residency programs now focus on preparing residents for academic careers with 1–2 years of research endeavors.

Although secondary degrees, specifically medical physician-scientist training programs, are considered a gateway to a successful academic career, a secondary degree was without statistical significant difference for R01 amount awarded. Contrary to prior studies, we did not find a gender disparity between average R01 monies in the specialty of neurosurgery. We found that males accounted for 91.36% of neurosurgeon grant recipients whereas females accounted for the remaining 8.64%. However, as of 2017, 91.6% of United States neurosurgeons were male and 8.4% were female [10]. Certainly, there is a well-known gender disparity for neurosurgeons within the United States; however, these data do not suggest a discrepancy between gender and R01 grant funding in the specialty of neurosurgery. Rather, female neurosurgeons acquired R01 grant funding proportional to their representation in the field of neurosurgery. Indeed, males accounted for 93.99% R01 monies; however, no statistically significant difference between average amount awarded and gender was identified. Factors based on our data that correlated to a statistically significant difference in amount of R01 monies awarded were between type of professorship and magnitude of h - index. This difference was expected as full professors have decades to advance their career, develop their publication profile, and become competitive applicants for R01 grant monies compared to physician-scientists beginning their academic careers. Moreover, high h – indexes by definition have more publications and citations to support their research endeavors and, ultimately, acquire R01 grant monies.

There are several limitations to the present study. The authors cannot comment on data preceding 2008 as this was not included in the cohort. The number of applications or rejections received by the NIH is not available and could not be included in the analyses. As such, the number, if any, of osteopathic neurosurgeons that applied for R01 funding during this timeframe cannot be commented on. We cannot comment on funding differences between minority groups as such data remains unavailable. The authors also relied on the NIH and each R01 grantee's institutional website to acquire data as well as Scopus to acquire the h – index for each author. Inaccuracies of any of these sources may alter the current data and statistical analyses. Moreover, the h – index is a representation of academic progress at a single point in time. As such, there is no way to ensure the h – index is up to date or a current representation of each academic neurosurgeon's publication achievements. Moreover, type of professorship at the time of R01 grant acquirement was based upon each neurosurgeon's biographical data on their institution's website, which may not necessarily be up to date.

## 5 Conclusions

The present study, to our knowledge, is the first to investigate if certain innate and acquired factors of the academic neurosurgeon correlate to successful R01 awards and amount of R01 monies awarded. Our analyses demonstrated a medical degree disparity for academic neurosurgeons who receive an R01 grant; however, a gender disparity was not identified. Factors identified to correlate to a statistically significant higher amount of R01 monies awarded included type of professorship and magnitude of h – index.

## Provenance and peer review

No commissioned, externally peer reviewed.

## Funding sources

This research did not receive any specific grant from funding agencies in the public, commercial, or not-for-profit sectors.

## Author contributions

JC and MW: primary authors of manuscript. JC, BK, AK, HG, EG, MB, LA, JE, EM, and MW: provided substantial contributions to the conception and design of the manuscript. JC, BK, AK, HG, EG, MB, LA, JE, EM, and MW: data acquisition and statistical analyses. JC, BK, AK, HG, EG, MB, LA, JE, EM, and MW: contributed to manuscript, read and approved the submitted version. JC, BK, AK, HG, EG, MB, LA, JE, EM, and MW: agree to be accountable for all aspects of the work ensuring that questions related to the accuracy or integrity of any part of the work are investigated and resolved.

## Declaration of competing interest

The authors have no conflicts of interest to declare.
